# The prospective sonographic assessment of the lower uterine segment after cesarean section and its clinical utility

**DOI:** 10.1007/s00404-025-07963-2

**Published:** 2025-02-01

**Authors:** Stephan Spahn, Alex Horky, Dianita Sugiyo, Franz Bahlmann, Ammar Al Naimi

**Affiliations:** 1 Department of Obstetrics and Gynecology, Buergerhospital - Dr. Senckenberg Foundation, Frankfurt Am Main, 60318 Frankfurt, Hessen, Germany; 2 Clinic for Gynecology and Obstetrics, Medical Faculty, Philipps University of Marburg, Marburg, Germany; 3 Department of Obstetrics and Prenatal Medicine, Goethe University Hospital of Frankfurt, Hessen, Germany

**Keywords:** Cesarean section, Sonography, Lower uterine segment, Vaginal birth after cesarean

## Abstract

**Purpose:**

This study aims to investigate the sonographic features of the lower uterine segment (LUS) and their association with successful vaginal birth after a cesarean section (VBAC).

**Methods:**

This is a prospective observational cohort study of women who underwent a first cesarean section (CS). Inclusion criteria were age over 18 years and open family planning. Women with a history of any additional uterine surgery, as well as a vertical or inverted T uterotomy during the CS were excluded. A pregestational transvaginal sonography, followed by serial transabdominal ultrasound examinations at the first, second, and third trimesters upon starting a new pregnancy were performed. Each intra-gestational examination involved measuring the LUS on a sagittal plane over a length of 3 cm starting from the most inferior identifiable part of the myometrium behind a full bladder. Logistic regression was performed to test the association between measures of the LUS and successful vaginal birth after CS.

**Results:**

96 women with a follow-up pregnancy within 2 years of the initial CS were included in the analysis. The pregestational RMT ratio was 62% and 38 (39%) women had a niche. The median thickness of the lower uterine segment was 8.34 mm (5.57–9.77), 4.75 mm (4.02–5.95), and 2.55 mm (2.01–3.55) at the first, second and third trimester, respectively. 70 women attempted VBAC and the risk of unplanned repeat CS was 37.1%. One millimeter increase in LUS thickness in the first trimester increased the odds of VBAC by 50–120% depending on the used measure (p < 0.05). This association weakened with increasing gestational age and the *p* values increased above 0.05.

**Conclusion:**

There is a good chance of successful VBAC for women attempting it. The sonographic assessment of the lower uterine segment during pregnancy could be helpful in counseling these women, albeit it seems that performing ultrasound during the first trimester is more informative than second and third trimesters.

## What does this study add to the clinical work

**Table:** 

The lower uterine segment sonography shows utility in predicting successful vaginal birth after caesarean section, albeit increasing gestational age at examination could limit the clinical value.	

## Introduction

About one-fifth of newborns are delivered with a cesarean section (CS) globally, and the rates of CS have been continuously increasing for the past 3 decades. Moreover, CSs are projected to account for one-third of global deliveries in 2030 [1]. CS provides an essential tool for improving perinatal fetal and maternal outcomes, and is a key part of the World Health Organization (WHO) obstetric care package [2]. Nevertheless, there is evidence that a high CS rate above 15% is an indicator of CS overuse beyond medical necessity and is unlikely to improve perinatal outcomes [3].

The operative maternal morbidity associated with CS remains low with a downward trend, and therefore, the procedure is considered safe by both women and physicians [4]. However, women with a history of CS are faced with an increased risk of long-term complications including bleeding disorders, pelvic pain, subfertility, and placenta accreta spectrum in a subsequent pregnancy [5, 6]. Uterine rupture and dehiscence are other major concerns for pregnant women attempting vaginal birth after cesarean section (VBAC) [7, 8], and cause around 30% of repeat CS in Germany [9].

Timely identification of women at extremely high risk of uterine rupture and accordingly discouraging a risky VBAC improves maternal health. Ultrasound seems to have some utility in predicting safe or successful VBAC [10]. Antenatal sonographic evaluation of the lower uterine segment (LUS) seems to be predictive of uterine dehiscence or rupture during trial of VBAC, but the clinical applicability needs to be assessed with prospective studies [11].

Published literature features conflicting findings in regard to LUS cutoff points reflective of sonographic measurements that are safe for a trial of VBAC [12]. This heterogeneity could arise from different methods of evaluating the full thickness of the LUS or strictly measuring the myometrial layer. Moreover, the timing of sonographic evaluation could significantly contribute to this heterogeneity. These findings highlight the importance of standardized techniques for LUS assessment [13].

The main aim of this study is to assess the sonographic characteristics of the post-cesarean LUS and its changes during a subsequent pregnancy and investigate the association of the sonographic measures with successful VBAC.

## Methods

This is a prospective observational cohort study investigating the pre- and the intra-gestational assessment of the lower uterine segment after experiencing a CS in a previous pregnancy. Patients who underwent a first CS in a tertiary center were invited to participate in the study before being discharged. Inclusion criteria were age over 18 years and open family planning, whereas the exclusion criteria were completed family planning, a history of any additional uterine surgeries (such as myomectomies) besides the CS, as well as a vertical or inverted T uterotomy during the CS.

**Fig. 1:**
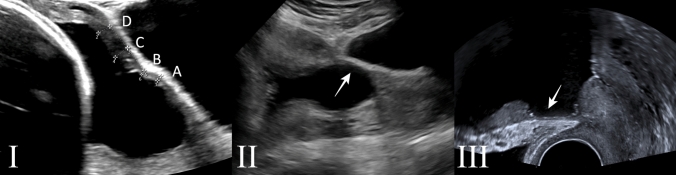
Ultrasound imaging showing: (**I**) the assessment of the lower uterine segment according to the study protocol at A, B, C, and D. (**II**) Transabdominal view of a bulging SC scar dehiscence (arrow). (**III**) Transvaginal view of a CS scar dehiscence with myometrial thinning (arrow)

Eligible and consenting women underwent the initial transvaginal sonographic examination 9–18 months post-surgery to measure the residual myometrial thickness (RMT) and assert the presence of a niche. RMT ratio was calculated as a percentage of RMT to the thickness of the entire anterior uterine wall. Furthermore, serial transabdominal ultrasound examinations at the first, second, and third trimesters were performed upon starting a new pregnancy within a year of the initial assessment. Each of the intra-gestational examinations involved three repetitive measurements of the lower uterine segment on the sagittal plane over a length of 3 cm starting from the most inferior identifiable part of the myometrium behind a full bladder. The myometrium was identified as a relatively hypoechogenic layer between two whitish hyperechogenic lines of the peritoneum and the chorio-amniotic membrane. The myometrial thickness at four points (from caudal to cranial A, B, C and D), each 1 cm apart, was measured and the average of these four measurements was used to estimate the thickness of the LUS. Moreover, the bladder volume was measured, and abnormal findings of lower uterine bulging were recorded and additionally assessed with transvaginal ultrasound as shown in Fig. 1.

The sonographic evaluations were conducted with transabdominal 3–9 MHz wideband convex transducers (C2-9-D) and transvaginal 5–13 MHz micro-convex transducers (GE RIC6-12-D) (Voluson E10, GE Healthcare GmbH, Munich, Germany). All images were saved on the institutional storage system (DICOM) and the electronic medical records were utilized to obtain the demographic data as well as the follow-up and pregnancy outcome measures for all patients.

Intra-class correlation coefficients (ICC) were calculated to quantify the intra-observer reproducibility of the uterine segment measurements. ICC values above 0.8 represent good agreement, whereas absolute agreement corresponds to higher values closer to 1. Continuous variables were reported in medians and interquartile ranges, and logistic regression was performed to test the associations with binomial outcomes. All statistical analyses were conducted with Stata® (ver. 18.0, Texas, USA). This study was approved by the Ethical Committee of the Hessen Regional Medical Council (Reg. Nr. 2019–1138-evBO).

## Results

Out of 150 women who were enrolled into the study and attended the first pregestational assessment, a total of 96 women entered and successfully finished a new pregnancy to be included in the analysis. The time between the first CS and the first examination was 13 [11–16] months and all women analyzed in this study got pregnant within 12 months of the initial examination. The demographic characteristics of the study cohort as well as their outcomes from the follow-up pregnancy are summarized in Table 1.

**Table 1:** Characteristics of the study cohort from the pre-enrollment and the follow-up pregnancies. IQR: interquartile range; n: number; CTG: cardiotocography

	Characteristic	Median (IRQ)/n (%)
Pre-enrollment pregnancy	Age in years	32 (29–36)
	Body mass index	27.8 (25.1–31.2)
	Gravidity	1 (1–2)
	Parity	1 (1–1)
	*Type of performed cesarean section*	
	Elective	32 (34.4%)
	Unplanned	59 (63.4%)
	Emergency	2 (2.2%)
	*Indications for the 1st cesarean section*	
	Psychological	2 (2.1%)
	Breech	25 (26.05%)
	Abnormal CTG tracing	18 (18.75%)
	Cervical dystocia	27 (28.1%)
	Preeclampsia-related	6 (6.25%)
	Other	18 (18.75%)
	Gestational age at delivery in weeks	39 (38–41)
Follow-up pregnancy	Bladder volume during exam in milliliter	265 (129–420)
	*Placental position*	
	Fundal	11 (11.5%)
	Anterior wall	55 (57.3%)
	Posterior wall	29 (30.2%)
	Placenta previa	1 (1%)
	*Cause of a repeat cesarean section both elective and unplanned (n: 52)*	
	Choice	14 (26.9%)
	Breech	5 (9.6%)
	Abnormal CTG tracing	4 (7.7%)
	Cervical dystocia	16 (30.8%)
	Suspicion of dehiscence	5 (9.6%)
	Other	8 (15.4%)
	Gestational age at delivery in weeks	40 (39–41)
	Birth weight in grams	3515 (3205–3810)
	APGAR score	10 (10–10)
	Arterial pH	7.28 (7.21–7.32)
	Uterine rupture	1 (1.04%)
	Dehiscence	4 (4.17%)

**Fig. 2:**
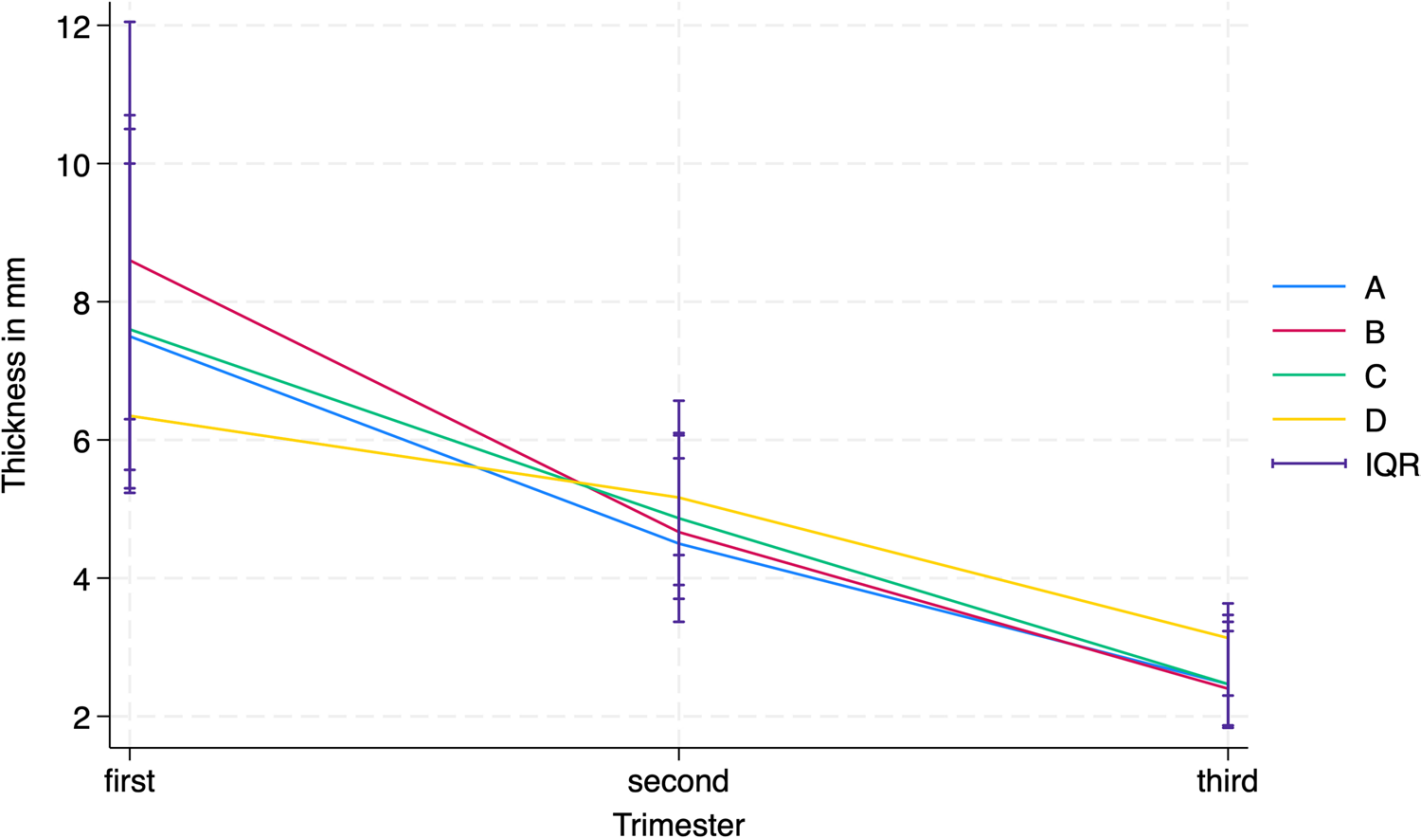
Two-way plot of the medians and interquartile ranges for the intra-gestational sonographic measurements at A, B, C, and D during the first, second and third trimesters

The pregestational RMT ratio was 62% and 38 (39%) women displayed the formation of a niche. The myometrial thickness showed a tapering trend from cranial (D) to caudal (A) and the interquartile ranges for all points narrowed with the progress of the pregnancy as shown in Fig. 2.

The measurement of the myometrial thickness at all points showed high reproducibility with an ICC above 0.8 irrespective of the trimester. The only exception was D during the second trimester with ICC of 0.54. The ICCs for these measurements are summarized in Table 2.

**Table 2:** Summary of the intra-class correlation coefficients (ICC) for the intra-observer reproducibility of measuring A, B, C, and D during the first, second and third trimesters

Measure	ICC (95% confidence interval)			
		First trimester	Second trimester	Third trimester
A	Individual	0.8641 (0.7418, 0.9385)	0.9363 (0.9016, 0.9606)	0.8845 (0.8418, 0.9181)
	Average	0.9502 (0.8960, 0.9786)	0.9778 (0.9649, 0.9865)	0.9583 (0.9410, 0.9711)
B	Individual	0.9568 (0.9125, 0.9812)	0.9459 (0.9166, 0.9665)	0.8932 (0.8534, 0.9244)
	Average	0.9852 (0.9690, 0.9936)	0.9813 (0.9705, 0.9886)	0.9617 (0.9458, 0.9735)
C	Individual	0.9791 (0.9571, 0.9910)	0.9355 (0.9004, 0.9601)	0.8224 (0.7607, 0.8724)
	Average	0.9929 (0.9853, 0.9970)	0.9775 (0.9644, 0.9863)	0.9329 (0.9051, 0.9535)
D	Individual	0.9728 (0.9433, 0.9885)	0.2866 (0.1161, 0.4669)	0.8719 (0.8248, 0.9091)
	Average	0.9908 (0.9804, 0.9961)	0.5465 (0.2827, 0.7243)	0.9533 (0.9339, 0.9678)

**Fig. 3:**
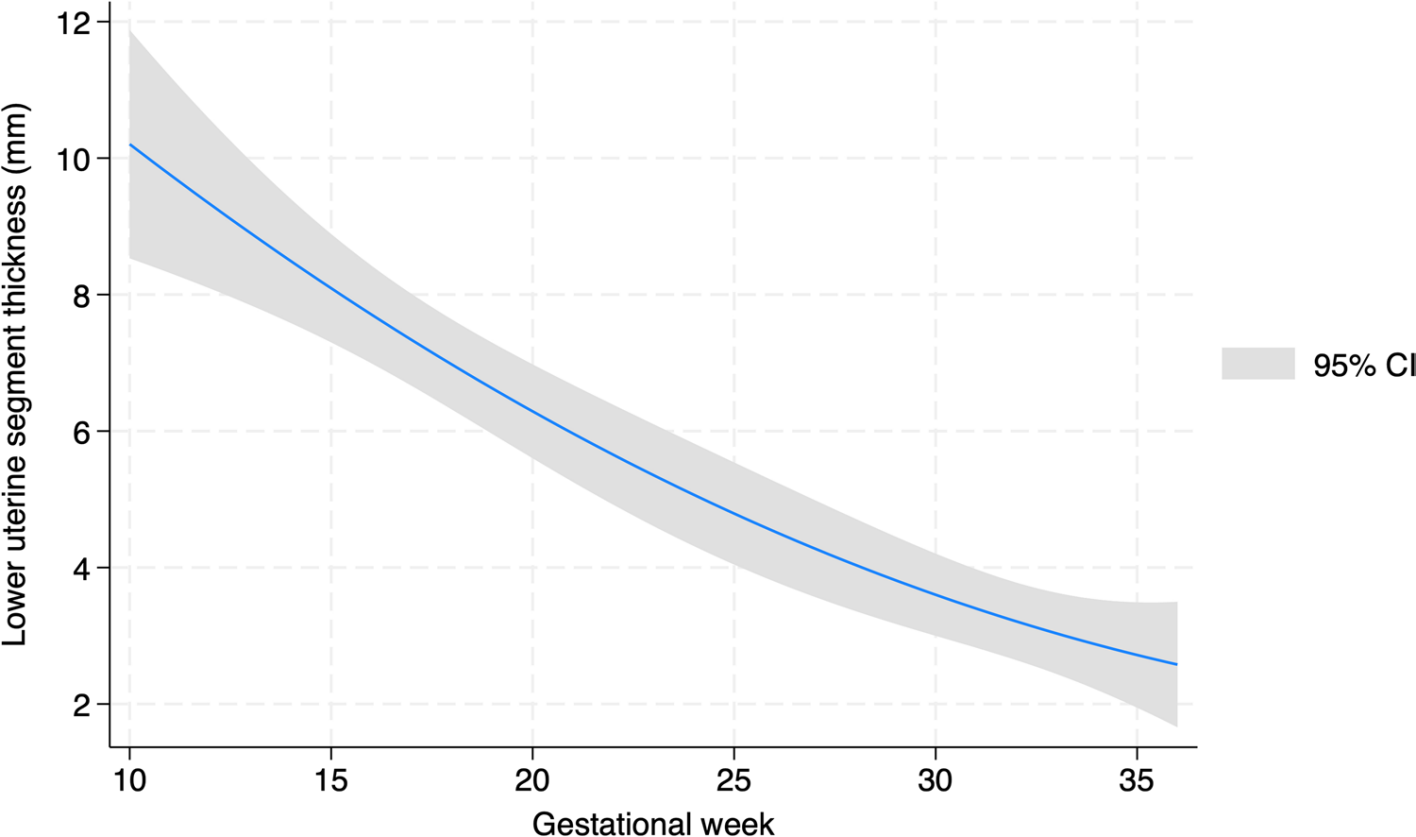
Two-way plot of the estimated lower uterine segment thickness throughout pregnancy. CI: confidence interval

The median thickness of the lower uterine segment was 8.34 mm (5.57–9.77), 4.75 mm (4.02–5.95), and 2.55 mm (2.01–3.55) at the first, second and third trimesters, respectively, and showed a similarly narrowing range with the increasing gestational age as shown in Fig. 3. The pregestational and intra-gestational measurements are summarized in Table 3.

**Table 3:** Summary of the study’s sonographic measurements. IQR: interquartile range; RMT: residual myometrial thickness; mm: millimeter

Variable	Median (IQR)		
Pregestational RMT%	0.62 (0.54, 0.76)		
	First trimester (mm)	Second trimester (mm)	Third trimester (mm)
A	7.5 (6.30, 10.50)	4.5 (3.36, 5.73)	2.4 (1.86, 3.36)
B	8.6 (5.56, 12.05)	4.66 (3.7, 6.56)	2.4 (1.86, 3.23)
C	7.6 (5.23, 10.7)	4.86 (3.9, 6.1)	2.46 (1.83, 3.46)
D	6.35 (5.3, 10)	5.16 (4.33, 6.06)	3.13 (2.3, 3.63)
Lower uterine segment	8.34 (5.57, 9.77)	4.75 (4.02, 5.95)	2.55 (2.01, 3.55)

**Fig. 4:**
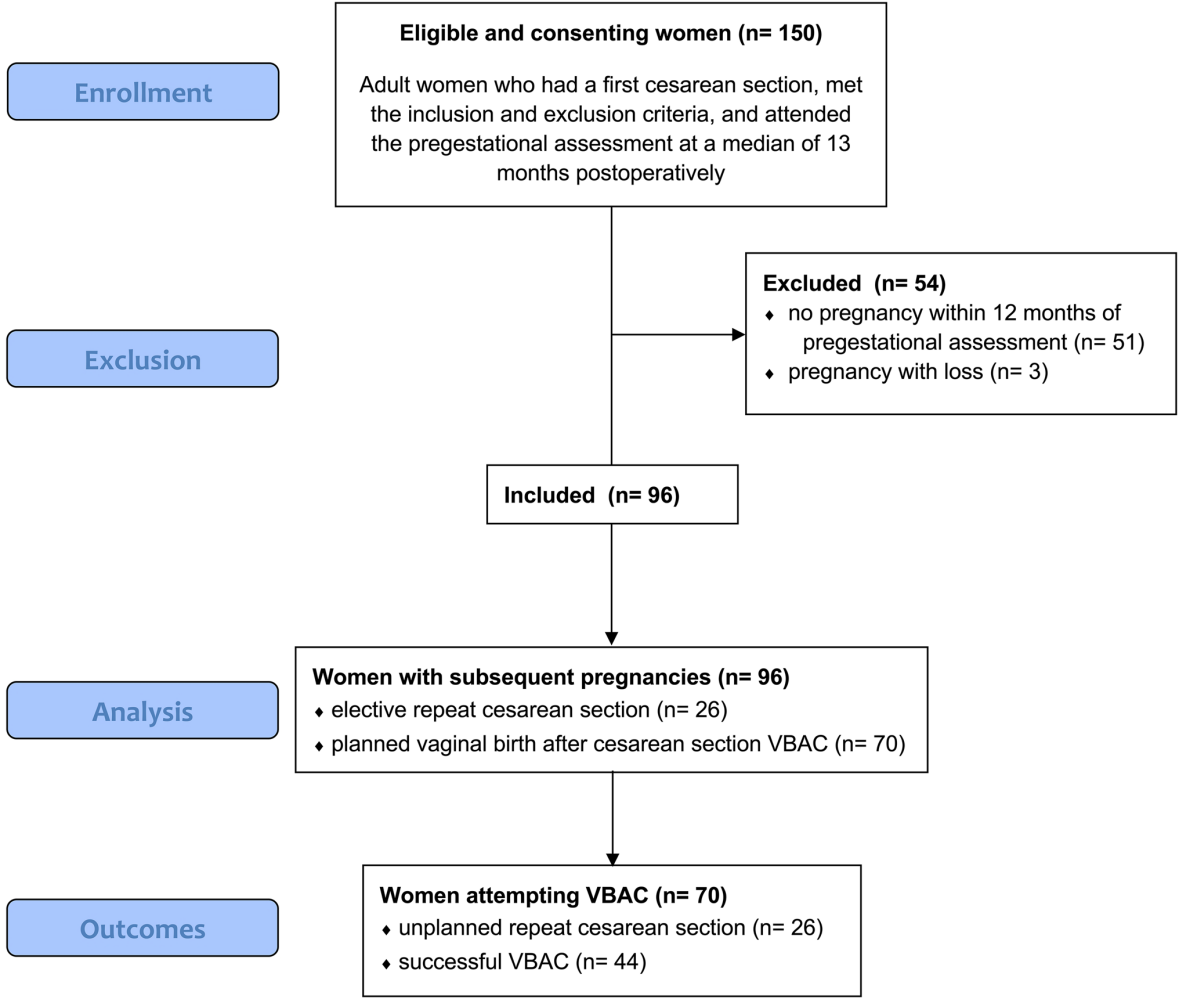
CONSORT flow diagram of the study participant

Twenty-six women (27.1%) had an elective repeat CS for several reasons, 5 of which were due to a sonographic suspicion of SC scar dehiscence due to bulging. Only 4 of those (80%) were intraoperatively confirmed and 1 (20%) was false positive. There was no statistically significant difference in the median lower uterine thickness between women with bulging (8.2, 5, and 2.7 mm) and women without bulging (9.4, 5.6, and 3.1 mm) in the first, second and third trimesters with *p* values of 0.75, 0.63 and 0.7, respectively. The risk of an unplanned repeat CS in the cohort of women who attempted VBAC was 37.1% and only one woman suffered an emergency CS due to uterine rupture. A study flow-chart with mode of delivery is provided in Fig. 4.

Univariate logistic regression showed a statistically significant association between the LUS thickness during the first trimester and the odds of achieving successful VBAC once this is attempted. One millimeter increase in thickness in the first trimester increased the odds of VBAC by 50–120% depending on the used measure. This association decreased with increasing gestational age and became statistically insignificant with *p* values above 0.05. Neither the pregestational RMT ratio nor the presence of a niche was associated with the odds of successful VBAC. The results of logistic regression for the association between all measures and VBAC are presented in Table 4.

**Table 4:** Results of univariate logistic regression for the association between the sonographic findings and the odds ratio of successful VBAC among women attempting it. CI: confidence interval; RMT: residual myometrial thickness

	Independent variable	Odds ratio (95% CI)	*P* value
Pregestational	RMT%	0.72 (0.06, 8.49)	0.79
	Niche formation	1.24 (0.23, 6.62)	0.80
First trimester	A	2.05 (1.04, 4.03)	0.04
	B	1.57 (1.06, 2.32)	0.02
	C	1.86 (1.08, 3.22)	0.03
	D	1.63 (0.79, 3.39)	0.19
	Lower uterine segment	2.25 (1.13, 4.49)	0.02
Second trimester	A	1.22 (0.97, 1.54)	0.08
	B	1.58 (1.15, 2.16)	0.004
	C	1.67 (1.17, 2.40)	0.005
	D	1.18 (0.10, 1.40)	0.05
	Lower uterine segment	1.48 (1.12, 1.95)	0.006
Third trimester	A	1.29 (0.98, 1.70)	0.07
	B	1.26 (0.95, 1.68)	0.11
	C	1.35 (0.92, 1.99)	0.13
	D	1.31 (0.93, 1.85)	0.13
	Lower uterine segment	1.35 (0.94, 1.93)	0.11

## Discussion

This prospective observational study was successful in obtaining complete follow-up data on all included 96 women after enrollment and exclusion. This reduced the risk of information bias caused by loss to follow-up, and thereby increased the validity of the study results led by the design [14]. The heterogeneity of measuring techniques is one of the major hindrances facing the utility of LUS ultrasound for predicting obstetrical outcomes after CS. Some studies systematically compared several techniques and showed that two-dimensional transvaginal ultrasound is the most reproducible [15]. Other studies showed very high interclass correlation between transvaginal and transabdominal ultrasound measurements of the LUS [10]. Supported by this high correlation, we decided to opt for transabdominal ultrasound in our study to decrease the invasiveness of the examination. Nevertheless, transvaginal ultrasound was performed in cases suspected of scar dehiscence to further verify or dismiss the suspicion. Moreover, the study followed and adhered to a pre-published measurement protocol to avoid problematic methodology and assess the myometrial thickness over 3 cm segment [16]. We demonstrated that the sonographic measurement of the LUS is reproducible with high intra-class correlation. This finding is supportive of previous longitudinal studies assessing the myometrial thickness at the CS scar throughout pregnancy. One specific study showed similar reproducibility with correlation coefficients around 0.86 [17]. Therefore, it is reasonable to utilize transabdominal ultrasound as a valid reproducible tool in assessing the myometrium throughout pregnancy.

Longitudinal data of the residual myometrial thickness showed a gradual decrease from the first to the third trimester [18]. Our study confirms this linear trend of myometrial thinning with increasing gestational age. This finding is critical in the discussions around an optimal cutoff value of the myometrial thickness that could be considered safe for VBAC. The heterogeneity of published literature is very likely to be influenced by the timing and gestational age of the sonographic assessment. Moreover, the interquartile range of the myometrial thickness narrowed with gestational age. This decrease in range reduces the variability of observations and affects the ability to detect differences for clinical utility. We are making this assumption supported by the weakening association between the sonographic thickness of the LUS with increasing gestational age as well as the gradual loss of statistical significance. Therefore, based on our findings, we would recommend assessing the LUS for the likelihood of successful VBAC during the first trimester. Integrating this information early into the antenatal care of women with a history of CS allows abundant time for counseling, planning the delivery, and fits within the inverted pyramid of prenatal care [19]. Women with a history of CS have been given special consideration within the recently published German–Austrian–Swiss guidelines for first trimester screening. There was a unanimous consensus that this cohort should undergo screening for CS scar pregnancies and placental anomalies for timely diagnosis of placenta accreta spectrum and vasa previa [20]. Incorporating our findings and simultaneously measuring the LUS thickness during first trimester screening could enhance the clinical utility of this recommendation by predicting VBAC success.

Interestingly, there was no difference in myometrial thickness among women with and without bulging. This is contradictory to other investigators showing a strong association between uterine dehiscence and LUS thickness. The methodology of the sonographic LUS measurement conducted by Bujold et al. included the bladder wall, and therefore, it is not exactly comparable to our study. This methodological heterogeneity could be the cause of discovering contradictory results [21]. Moreover, not all women with bulging are women with dehiscence. Despite the conflicting findings, our study, as well as Bujold et al., show the diagnostic utility of ultrasound in identifying scar dehiscence. The bulging of the LUS is highly specific for dehiscence with 80% specificity, and we propose utilizing it as a sonographic sign that identifies women with high risk for uterine rupture.

Neither the presence of a niche nor the pregestational RMT was associated with a successful VBAC. The pregestational sonographic assessment of CS scars should not be recommended based on this finding. However, the importance of non-gestational CS scar evaluation withholds even without an association with the obstetrical outcomes. Women with a history of CS could benefit from a sonographic evaluation especially if they were suffering from post-cesarean dysmenorrhea or menorrhagia [22]. Aside from showing the utility of ultrasound in assessing women with a history of CS, this study displays the safety and high success rate of VBAC for women attempting it. Two out of three women were able to achieve VBAC at a risk of 1.4% for uterine rupture. Although the risk of rupture in our cohort is double the quoted risk of 0.7% in the literature due to our small sample size, the risk remains low without causing serious maternal morbidity or mortality. Therefore, VBAC should be offered to women with a history of CS entirely based on favorable epidemiological data, neonatal, and maternal outcomes [23].

Performing univariate logistic regression without adjusting for individual patients’ demographic could be considered a limitation of our study. The aim of our study was to investigate the utility of the sonographic measures irrespective of the demographic characteristics. Therefore, univariate analysis was deemed appropriate. Multivariate analysis is essential for building predictive models, which would be the natural follow-up required to utilize our study findings similar to other studies [21]. Nevertheless, predictive models do not uniformly perform in different populations. Thus, we do not assume that a predictive model based on our data would be appropriate for general use [15].

In conclusion, pregnant women with a history of CS should be assured that attempting VBAC is safe. The likelihood of successful VBAC is high and the sonographic assessment of the lower uterine segment during pregnancy could be helpful in predicting the success with increasing myometrial thickness. More importantly, ultrasound finding of bulging is specific in detecting women with dehiscent CS scars, who could be at high risk of uterine rupture.

## Data Availability

No datasets were generated or analysed during the current study.

## Code availability

Non-applicable.
